# Oral Yeast Colonization and Fungal Infections in Peritoneal Dialysis Patients: A Pilot Study

**DOI:** 10.1155/2017/4846363

**Published:** 2017-12-21

**Authors:** Liliana Simões-Silva, Sara Silva, Carla Santos-Araujo, Joana Sousa, Manuel Pestana, Ricardo Araujo, Isabel Soares-Silva, Benedita Sampaio-Maia

**Affiliations:** ^1^i3S-Instituto de Investigação e Inovação em Saúde, Universidade do Porto, Porto, Portugal; ^2^INEB-Instituto de Engenharia Biomédica, Universidade do Porto, Rua Alfredo Allen 208, 4200-180 Porto, Portugal; ^3^Faculty of Medicine, University of Porto, Porto, Portugal; ^4^Faculty of Dental Medicine, University of Porto, Porto, Portugal; ^5^Department of Nephrology, São João Hospital Center, EPE, Porto, Portugal; ^6^Department of Physiology and Cardiothoracic Surgery, Cardiovascular R&D Center, Faculty of Medicine, University of Porto, Porto, Portugal; ^7^Department of Renal, Urological and Infectious Diseases, Faculty of Medicine, University of Porto, Porto, Portugal; ^8^Ipatimup, Institute of Molecular Pathology and Immunology of the University of Porto, Porto, Portugal; ^9^Department of Medical Biotechnology, School of Medicine, Flinders University, Adelaide, SA 5042, Australia

## Abstract

Peritonitis and exit-site infections are important complications in peritoneal dialysis (PD) patients that are occasionally caused by opportunistic fungi inhabiting distant body sites. In this study, the oral yeast colonization of PD patients and the antifungal susceptibility profile of the isolated yeasts were accessed and correlated with fungal infection episodes in the following 4 years. Saliva yeast colonization was accessed in 21 PD patients and 27 healthy controls by growth in CHROMagar-Candida® and 18S rRNA/ITS sequencing. PD patients presented a lower oral yeast prevalence when compared to controls, namely, *Candida albicans*. Other species were also isolated, *Candida glabrata* and *Candida carpophila*. The antifungal susceptibility profiles of these isolates revealed resistance to itraconazole, variable susceptibility to caspofungin, and higher MIC values of posaconazole compared to previous reports. The 4-year longitudinal evaluation of these patients revealed *Candida parapsilosis* and *Candida zeylanoides* as PD-related exit-site infectious agents, but no correlation was found with oral yeast colonization. This pilot study suggests that oral yeast colonization may represent a limited risk for fungal infection development in PD patients. Oral yeast isolates presented a variable antifungal susceptibility profile, which may suggest resistance to some second-line drugs, highlighting the importance of antifungal susceptibility assessment in the clinical practice.

## 1. Introduction

Peritoneal dialysis (PD) is a home-based and widely used renal replacement therapy for patients with end-stage renal disease (ESRD). In PD patients, infectious complications, namely, peritonitis and exit-site infections, account for a significant percentage of catheter loss, transfer to haemodialysis, prolonged hospitalization, or even death, making prevention of infection a critical step to the success of a PD program [1]. Although a rare event, fungal peritonitis is associated with significant morbidity and mortality in PD patients [2]. Fungal exit-site infections are more frequent than peritonitis, but more easily resolved, although they may potentiate the development of a subsequent peritonitis [3]. Fungal infections are primarily caused by opportunistic fungal pathogens, such as *Candida* species, that take advantage of a locally or systemically debilitated immune system to proliferate in the human host and cause disease. In a recent study where fungal exit-site infections of PD patients were evaluated, the most frequently isolated species were *Candida parapsilosis* (67%), followed by *Candida glabrata* (10%), *Candida famata* (7%), and *Candida zeylanoides* (7%) [4].

Factors that influence the occurrence of PD-related infections are still not completely understood. Some authors have highlighted the importance of the oral microbiome as a starting point for dissemination of pathogens to distant body sites [5, 6]. Despite the association between oral pathology and adverse outcomes of renal patients [7], so far, existing studies have neither evaluated the oral yeast colonization of PD patients nor evaluated its relation with the development of fungal infections. This topic is particularly relevant when considering the opportunistic character of *Candida* species, a frequent colonizer of the oral cavity [6] and the immune impairment of ESRD patients and knowing that chronic kidney disease itself, and PD therapy in particular, alters significantly the oral milieu [8].

Therefore, in the present study, the oral yeast colonization and the oral health of PD patients were characterized and compared with a healthy population. The antifungal susceptibility profile of yeasts isolated from the oral cavity was also assessed. Additionally, the clinical history of fungal infections was evaluated and related with the oral yeast colonization of PD patients.

## 2. Material and Methods

Patients followed up for at least 1 month in the PD outpatient clinic of the Nephrology Department of “Centro Hospitalar de S. João,” over 18 years of age and with no recent history of infection (less than 1 month) were invited to participate in the study. A convenience sample was obtained related with the attendance of patients to the outpatient clinic during a period of 6 months. A group of 21 PD patients accepted to participate and were included in the study. The control group consisted of 27 adult healthy subjects, including 10 PD family members (in order to select individuals living in similar environment and conditions as the patients) and 17 nonfamily members of PD patients. The exclusion criteria were: inability to give informed consent, pregnancy, and severe acute illness. The study protocol was approved by the Ethics Committee for Health and Institutional Review Board of “Centro Hospitalar de S. João,” and all recruited patients and controls were asked to give their written informed consent. This work is constituted by a cross-sectional study, regarding the comparison of *Candida* oral colonization in PD patients and controls, followed by a longitudinal study, in which the history of *Candida* spp. infections was analysed during 4 years to establish a comparison between the *Candida* species present in the oral cavity and the *Candida* species responsible for subsequent PD-related fungal infections. Regarding the longitudinal evaluation of PD-related fungal infections, the study began with 21 patients, and due to PD technique dropout, 20, 19, 14, and 11 patients remained at the end of the first, second, third, and fourth follow-up year, respectively.

Patients' clinical information was gathered including age, gender, smoking habits, blood pressure, aetiology of renal disease, residual renal function, PD vintage, infectious complications during PD, and PD-related fungal infection episodes and agents. Demographic information was gathered for control population, namely, age, gender, and smoking habits.

A noninvasive intraoral examination was performed in both groups in order to evaluate the oral hygiene by visible plaque index (VPI) in four sites of each tooth (mesiobuccal, midbuccal, distobuccal, and midlingual); the percentage of the examined sites with visible plaque ranged from 0% to 100%. Whole saliva was collected in both groups before oral examination for microbial analysis and pH evaluation. The patients were instructed not to eat, drink, and perform the normal mouth hygiene at least two hours before the procedure. Samples of nonstimulated saliva were collected in one time point for each patient under resting conditions. The patients were asked to spit the whole-mouth saliva after 5 min. The volume was quantified gravimetrically, and the salivary flow rate was determined (mL min^−1^). The pH of saliva was determined immediately after collection using pH strips (5.0–8.0, Duotest, Germany). The saliva was mixed 1 : 1 in Brain Heart Infusion with 20% glycerol and cryopreserved at −80°C until microbial analysis.

Saliva samples were unfrozen for yeast isolation and quantification. The samples were serially diluted with 0.9% sterile NaCl solution and plated in triplicate in a selective and differential culture medium, CHROMagar-Candida. Plates were incubated aerobically for 48 h at 37°C. Total number of colonies was determined, and quantification results were expressed in logarithmic scale of colony forming units per ml of saliva (Log_10_ CFU mL^−1^). Identification of *Candida albicans* was possible due to the specific colour of the colonies. Isolates were identified by 18S rDNA and internal transcribed spacer (ITS) region DNA sequencing approach as previously described [9]. PCR amplification was performed using a group of specific primers: EF3 (5′-TCCTCTAAATGACCAAGTTTG-3′), EF4 (5′-GGAAGGG[G/A]TGTATTTATTAG-3′), fung5 (5′-GTAAAAGTCCTGGTTCCCC-3′), ITS1 (5′TCCGTAGGTGAACCTTGCGG-3′), and ITS4 (5′-TCCTCCGCTTATTGATATGC-3′) in a Thermo-Hybaid-PX2 thermal cycler. Amplification products were visualized in a polyacrilamide gel followed by silver-staining. Sequence analysis was performed in a genetic analyser ABI-Prism-3100 (Applied Biosystems). Genomic data were compared with a database that comprises a large collection of yeast sequences of 18S rDNA and ITS regions obtained from GenBank.

Antifungal susceptibility testing was performed by the determination of minimum inhibitory concentration (MIC) and according to clinical breakpoints (CBP) defined in the M27-A3 and M27-S4 protocols of the Clinical and Laboratory Standards Institute (CLSI) (http://clsi.org/). Due to the loss of viability of some isolates, antifungal susceptibility was performed in 2 isolates from the PD group and 4 out of 10 from the controls. The following antifungals were tested: voriconazole (Pfizer, Groton, CT), posaconazole (Schering-Plough, Summit, NJ), fluconazole (Pfizer, Groton, CT), amphotericin B (Bristol-Myers Squibb, New York, NY, USA), caspofungin (Merck, Rahway, NJ, USA), anidulafungin (Pfizer, Groton, CT, USA), and micafungin (Astellas Pharma, Inc., Tokyo, Japan). For species whose clinical breakpoints are not defined, the phenotype was characterized based on epidemiological cutoff values (ECVs) according to Pfaller and Diekema [10].

Saliva biochemical parameters were quantified by an automatic analyser, Pentra C200 (Horiba ABX Diagnostics, Switzerland). In brief, phosphate was detected by UV using phosphomolybdate, whereas α-amylases were detected by an enzymatic photometric test, using the substrate 4,6-ethylidene-(G7)-p-nitrophenyl-(G1)-α-D6 maltoheptaoside (EPS-G7). In addition, salivary IgA was determined by immunoturbidimetry and urea by enzymatic UV test (method “Urease–GLDH”).

Statistical analyses were performed using IBM® SPSS® version 23.0 (Statistical Package for Social Sciences). The categorical variables were described through relative frequencies (%) and analysed by the chi-square independence test or Fisher exact test when more than 1 cell had expected counts less than 5. The normality test was performed with Shapiro-Wilk. When normally distributed, continuous variables were described using mean ± standard deviation (SD) and analysed by student's *t*-test, whereas when not normally distributed, continuous variables were described using median (min, max) and analysed by the Mann–Whitney *U* test. *P* < 0.05 was assumed to denote a significant difference.

## 3. Results

PD patients and controls presented similar demographic characteristics (Table 1).

The clinical history of PD patients, such as the most prevalent aetiologies of chronic kidney disease, and the most relevant clinical data, such as PD vintage, residual renal function, and blood pressure determined at the day of sample collection, are presented in Table 2. Additionally, this table also presents the prevalence of patients on specific medication reported to be associated with altered susceptibility to fungal infections, namely, calcium channel blockers, statins, vitamin D, and iron supplementation [11–14].

The study was initiated by an oral clinical evaluation and saliva collection. At this point, the average time on PD therapy was 15.5 ± 16.9 months, ranging from 1 to 72 months (Table 2).

PD patients presented a lower prevalence of yeasts in saliva compared to the healthy controls; however, the difference did not attain statistical significance (Table 3). Three *Candida* species were identified, namely, *C. albicans* and *C. glabrata* in PD patients and *C. albicans* and *C. carpophila* in controls. The prevalence of *C. albicans* was significantly lower in PD patients than in controls. One control was colonized by two different species: *C. albicans* and *C. glabrata*. Despite the low oral yeast prevalence in PD patients, the quantification of total yeast number (Log_10_ CFU mL^−1^) in individuals colonized with yeast did not differ between PD patients and the control group (Table 3).

Six *Candida* isolates from the oral cavity were analysed for antifungal susceptibility profile: 4 isolates from controls (3 *C. albicans*, and 1 *C. glabrata*) and 2 isolates from PD patients (1 *C. albicans*, and 1 *C. carpophila*). All the isolates were resistant to itraconazole (MIC > 1 µg mL^−1^); presented a non-wild type phenotype regarding posaconazole (NWT, MIC > 2 µg mL^−1^); and were susceptible to anidulafungin (MIC < 0.125 µg mL^−1^), voriconazole (MIC < 0.125 µg mL^−1^), and fluconazole (MIC < 4 µg mL^−1^). A similar susceptibility profile was obtained for all isolates regarding amphotericin B (MIC = 1 µg mL^−1^) and flucytosine (MIC = 0.125 µg mL^−1^). *Candida glabrata* isolated from the control group was the only *Candida* isolate resistant to micafungin (MIC = 0.5 µg mL^−1^). A variable susceptibility to caspofungin (MIC ranging from 0.25 to 1 µg mL^−1^) was observed for the *Candida* isolates. The susceptibility epidemiological cutoffs for antifungals are still not defined for *C. carpophila*; nevertheless, the susceptibility values were 0.125 µg mL^−1^ for flucytosine, 0.25 µg mL^−1^ for voriconazole, 0.5 µg mL^−1^ for amphotericin B, 1 µg mL^−1^ for caspofungin and micafungin, 2 µg mL^−1^ for posaconazole, and 4 µg mL^−1^ for fluconazole, itraconazole, and anidulafungin.

Table 4 depicts the oral factors that can play a role on yeast growth in the oral cavity. Saliva pH and urea levels were higher in PD patients when compared to the control group.

Regarding PD-related fungal infections, clinical records of this group of PD patients were analysed. In the period previous to sample collection, no peritonitis of fungal origin was recorded, and only one exit-site infection episode was recorded from fungal origin, namely, due to *C. parapsilosis*. This patient, however, did not present yeast oral colonization at the time of the study (approximately one year after the infection).

Moreover, concerning the longitudinal evaluation, PD-related infections of fungal origin were recorded during the 4 years following sample collection. During this period, 4 exit-site fungal infection episodes were recorded, 2 of them in the same patient. These 2 episodes occurred with a time difference of more than 5 months and were caused by *Candida parapsilosis*, although in one of the episodes bacterial agents were also isolated. Other two patients presented infections either by *Candida parapsilosis* or *Candida zeylanoides*. No peritonitis of fungal origin was recorded for these patients within this time frame.

**Figure 1:**
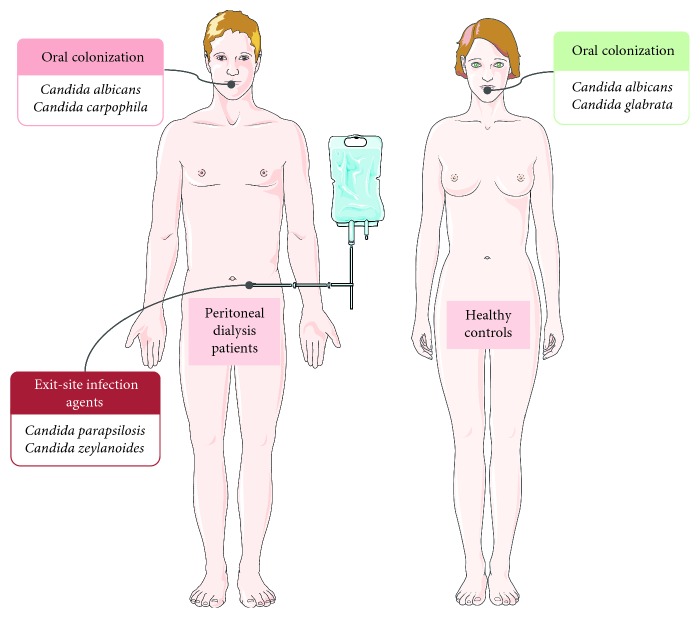
Oral *Candida* colonization of PD patients and healthy controls and *Candida* agents responsible for PD-related infections. Figure was produced using Servier Medical Art, http://www.servier.com/Powerpoint-image-bank.

A comparison between the *Candida* species identified in saliva of PD patients and fungal infectious agents responsible for the exit-site infections did not reveal the existence of common species (Figure 1).

In addition, none of the patients that presented oral yeast colonization developed PD-related fungal infections. Also, no relationship was found between PD-related fungal infectious agents and oral colonization of family controls.

## 4. Discussion

PD-related infections from fungal origin are important complications in PD patients, and the opportunistic fungi inhabiting distant body sites may represent a major source of infection. According to our results, oral yeast colonization constitutes a limited risk for fungal infections in PD patients, due to the lack of relationship between fungal oral colonizers and PD infectious agents. Additionally, PD patients presented a lower prevalence of oral yeasts, in particular *C. albicans*, in comparison to a healthy population.

Interestingly, non-*Candida albicans* species were also found to be normal colonizers of saliva, namely, *C. carpophila* in PD patients and *C. glabrata* in controls. To our knowledge, this is the first study to detect the yeast *C. carpophila* in human saliva. It is possible that the modified oral environment of PD patients results in a shift of yeast prevalence compared with the healthy population resulting in the emergence of rare yeasts, such as *C. carpophila*.

Several factors may contribute to the altered prevalence of yeasts in the oral cavity of PD patients, in particular regarding *C. albicans*. *C. albicans* is the most prevalent fungal specie in the oral cavity, being described as more sensitive than other *Candida* species to potential combined environmental factors present in the oral cavity [15]. Thus, the lower oral *C. albicans* colonization of PD patients could be justified not only by a higher exposure to antifungal therapy, recommended during an antibiotic course [16, 17], but also by alterations of the oral environment secondary to systemic ESRD effects, PD therapy, and medication. Regarding the antifungal therapy, the protocol followed in our department consists in the prescription of fluconazole (100 mg day^−1^) whenever a patient starts antibiotic therapy after the first use without success. However, since none of our patients had an infectious episode in the month previous to sample collection, this may not be the reason for a lower level of *Candida albicans* colonization. Regarding the medication of these patients, several molecules are reported to be associated with altered susceptibility to fungal infections and can influence yeast colonization in this population. 47.6% of the PD patients were on calcium channel blockers therapy, described to have an inhibitory effect on oxidative stress response of *Candida albicans* [11]; 57.1% were prescribed with statins, 3-hydroxy-3-methylglutaryl-CoA (HMG-CoA) reductase inhibitors, used to lower patients' cholesterol but that also affects ergosterol levels exhibiting antifungal properties [12]; 71.4% were supplemented with vitamin D known to affect fungal growth in a dose-dependent manner [13]; and 90.5% had iron supplementation, an essential element for microbial growth and known to influence host susceptibility to *C. albicans* infections [14].

In order to investigate other possible causes for the reduced oral *C. albicans* colonization of PD patients, we further evaluated specific oral parameters such as oral hygiene, smoking habits, and saliva biochemistry, given that previous studies found relevant changes in the oral status of chronic kidney disease patients undergoing PD [18]. In accordance to previous studies, saliva pH and urea levels were significantly altered in PD patients in comparison with controls [8, 19]. The high urea levels may contribute in part to the higher salivary pH, due to the ammonia production as a result of urea hydrolysis [8, 19]. This altered oral pH may have an impact on *Candida* growth, given that oral *Candida* isolates have been shown to be more adapted to acidic conditions [20]. It is known that neutral to alkaline pH can cause severe stress to *C. albicans* including impaired nutrient acquisition, as a consequence of a disrupted proton gradient, and malfunctioning of pH-sensitive proteins [21]. In addition, changes in the oral pH may be the major ecological factor that alters the oral commensal microbiome, leading to shifts in its natural diversity [22]. Recent advances on bacterial–fungal interkingdom communication have shown a negative correlation between the *Candida* load and the diversity of the salivary microbiome [22]. This suggests that, globally, the oral microbiome of PD patients may be altered; the impact of these changes on PD-related infections deserves to be further clarified.

Despite the limited number of isolates tested for antifungal susceptibility profile, we verified that all were resistant to itraconazole and presented a non-wild type phenotype regarding posaconazole. Also, the MIC values of posaconazole for *C. albicans* and *C. glabrata* isolates are higher than the previously reported values for wild-type strains [23]. This resistance profile may not be associated to the frequent prophylactic antifungal therapy prescription during an antibiotic course [16, 17], given that oral nystatin and fluconazole are the common choices [1]. However, itraconazole and posaconazole susceptibility profiles in oral *Candida* isolates are a matter of concern since both these drugs are second-line agents for the treatment of oropharyngeal candidiasis [24]. Also, we observed variable susceptibility profiles of *Candida* isolates to caspofungin. Taking into account that itraconazole, posaconazole, and caspofungin are prescribed for the treatment of systemic fungal infections [25] and that previous studies report the existence of antifungal resistance to itraconazole and caspofungin [26], we consider it relevant to determine the susceptibility to these antifungals in all clinical isolates.

This study presents some limitations, particularly the limited number of patients analysed and the methodology for yeast isolation (direct spread plate technique), which is associated with limited sensitivity [27]. However, it is important to highlight that the percentage of *Candida* that we obtained is similar to other studies that used the same methodology [28]. On the other hand, it is important to highlight that 10 elements of the control group were family members of the PD patients. This is a relevant aspect since the oral microbial colonization is strongly correlated to the diet, oral hygiene, and familial predisposition.

In conclusion, oral yeast colonization may represent a limited risk for fungal infections in PD patients, given that in this pilot study there is an absence of relationship between patients with oral yeast colonization and the development of PD-related fungal infections; the *Candida* species found in oral cavity are different from the ones identified as PD-related fungal infectious agents; and also, PD patients present a low prevalence of oral yeast colonization, namely, *C. albicans*. Despite the low number of oral *Candida* isolates, the antifungal susceptibility profile revealed a possible resistance to some second-line drugs, suggesting the need for the assessment of antifungal susceptibilities in clinical practice. Further studies are still necessary to fully characterize the oral yeast colonization in this population.

## Figures and Tables

**Table 1:** Age and sex of peritoneal dialysis (PD) patients and controls.

	PD patients (*n* = 21)	Controls (*n* = 27)	*P* value
Age (years)	46.8 ± 9.7	43.2 ± 11.9	0.273
Sex (male, %)	42.9%	18.5%	0.066

Results are shown in prevalence (%) or mean ± SD. PD, peritoneal dialysis.

**Table 2:** Aetiology of chronic kidney disease (CKD), time on peritoneal dialysis, residual renal function, and blood pressure of peritoneal dialysis (PD) patients.

	PD patients (*n* = 21)
Aetiology of CKD	
Glomerular disease	52.3%
Diabetic nephropathy	19.0%
Other glomerular disease	33.3%
Tubulointerstitial disease	23.8%
Autosomal dominant polycystic kidney disease	14.3%
Other tubulointerstitial disease	9.5%
Unknown	23.8%
PD vintage (months)	15.5 ± 16.9
Residual renal function (mL min^−1^)	7.0 ± 4.5
Blood pressure	
Systolic	130.2 ± 19.7
Diastolic	78.3 ± 11.0
Therapy	
Calcium channel blockers	47.6%
Statins	57.1%
Vitamin D supplementation	71.4%
Iron supplementation	90.5%

Results are shown in prevalence (%). CKD, chronic kidney disease; PD, peritoneal dialysis.

**Table 3:** Prevalence and quantification of yeast colonizers in peritoneal dialysis (PD) patients and controls.

	PD patients (*n* = 21)	Controls (*n* = 27)	*P* value
Yeast prevalence	9.6% (2/21)	33.3% (9/27)	0.083
Yeasts (Log_10_ CFU mL^−1^)	2.39 ± 0.80	2.55 ± 0.82	0.803
Species prevalence			
*Candida albicans*	4.8% (1/21)	33.3% (9/27)	0.029^∗^
*Candida glabrata*	0%	3.7% (1/27)	>0.999
*Candida carpophila*	4.8% (1/21)	0%	0.438

Results are prevalence (%) or mean ± SD. PD, peritoneal dialysis; CFU, colony-forming units. ^∗^*P* < 0.05.

**Table 4:** Smoking habits, oral hygiene, and saliva biochemistry of peritoneal dialysis (PD) patients and controls.

	PD patients	Controls	*P* value
Smoking habits			
Past (%)	58.3%	40.9%	0.331
Present (%)	16.7%	22.7%	>0.999
Visual plaque index (%)	56 (16, 100)	69 (14, 100)	0.489
Saliva biochemistry			
Flow rate (mL min^−1^)	0.41 (0.05, 1.06)	0.26 (0.10, 1.04)	0.432
pH	8.0 (6.5, 8.0)	6.8 (6.2, 8.0)	<0.001^∗^
Urea (mg dL^−1^)	110.41 ± 36.64	47.85 ± 23.88	<0.001^∗^
Phosphorus (mg dL^−1^)	22.76 ± 8.19	18.58 ± 16.86	0.486
IgA (mg dL^−1^)	143.0 (126.0, 178.0)	136.5 (30.0, 167.0)	0.361
Amylase (U L^−1^)	309.45 (127.40, 757.50)	562.6 (312.90, 571.10)	0.310

Results are prevalence (%), median (min, max) or mean ± SD; PD, peritoneal dialysis. ^∗^*P* < 0.05.
